# Next Steps: Eliminating Disparities in Diabetes and Obesity

**DOI:** 10.5888/pcd12.150102

**Published:** 2015-05-07

**Authors:** Debra L. Haire-Joshu

In 2012, 29.1 million Americans, or 9.3% of the population, had diabetes, contributing an estimated $245 billion to US health care costs (1). Type 2 diabetes, which accounts for 90% or more of diabetes cases, affects people of all ages, economic groups, races, and ethnicities (1). However, certain populations, including American Indians, African Americans, and those with socioeconomic disadvantages, have a disproportionate burden of disease and associated complications (1). Despite evidence of effective means to prevent type 2 diabetes through obesity prevention and control, there remains a significant gap between research and practice in real world settings that limits impact. Translational research is needed to inform innovative strategies for preventing and controlling diabetes across high-risk groups.

The Washington University Center for Diabetes Translation Research (WU-CDTR) is 1 of 7 centers across the country funded by the National Institute of Diabetes and Digestive and Kidney Diseases (NIDDK) of the National Institutes of Health whose purpose is to enhance the efficiency, productivity, and effectiveness of diabetes translation research (2). The research is conducted in partnership with the National Congress of American Indians, an organization that represents 566 federally recognized tribal nations (3). The WU-CDTR supports transdisciplinary researchers committed to improving the quality, quantity, and effective translation of research to prevent diabetes and address obesity as a major cause across diverse populations. Researchers study the root causes of diabetes and the impact on translation of evidence-based interventions. These root causes are defined by multiple social constructs that influence health, including poverty, living and working conditions, housing quality, and access to healthy food and safe neighborhoods. Individual constructs, such as health literacy, communication barriers, or cultural differences, are also associated with diabetes disparities (4). Differential experiences and exposure to these dimensions of disparity across the life course contribute to the prevalence of obesity and diabetes among diverse racial and ethnic groups (5). An understanding of these influences is needed to guide implementation research to prevent and control diabetes.

Translational researchers need time to work together to ask questions and solve complex problems. The WU-CDTR and the Washington University Institute of Public Health cosponsored a 1-day conference for researchers, Next Steps: Eliminating Disparities in Diabetes and Obesity. In this context, a review of the 2011 NIDDK strategic plan (6) challenged us to “take stock” of our progress in addressing translational diabetes research relevant to the work of the WU-CDTR. We prioritized 4 questions to guide our review of research activities: 1) What is the influence of life course exposure to poor living environments on diabetes prevention and control? 2) How can structures and policies influence behavior change to prevent diabetes? 3) How can innovations in health communication science and technologies be advanced to test strategies for addressing diabetes disparities? 4) What are key practices for adapting culturally appropriate, evidence-based interventions to real-world settings while expanding reach?

The conference provided a forum for WU-CDTR researchers to discuss, critique, and identify methods for answering these provocative questions. Forty-three researchers representing 14 disciplines and 5 institutions across the country collaborated on a collection of 12 articles in *Preventing Chronic Disease* (*PCD*). These articles document research across various stages of development and inform implementation of evidence-based practices across high-risk populations. Although research on several topics in this collection is in its early stages, we anticipate that these articles will provide practitioners with evidence and leverage points for their efforts in controlling diabetes and obesity. The articles are identified in *PCD*’s table of contents as part of the collection and will be compiled into a single PDF download on the *PCD Collections *page shortly after release (www.cdc.gov/pcd/collections/index.htm). Articles in the collection cover 3 major topics: contextual risk factors, environment and policy issues, and the emerging evidence base for effective interventions.

Three articles on contextual risk factors address the influence of life course exposure to poor living environments on diabetes development and prevalence. Duncan et al (7) describe the link between child maltreatment and diabetes in young adulthood and their differential effects by sex in “Relationship Between Child Abuse and Neglect in Childhood and Diabetes in Adulthood: Differential Effects By Sex, National Longitudinal Study of Adolescent Health.” Marley and Metzger (8) describe the influence of neighborhood risk factors, poverty, and stress on diabetes outcomes in “A Longitudinal Study of Structural Risk Factors for Obesity and Diabetes among American Indian Young Adults, 1994–2008.” The impact of life course exposure to risk factors in the physical environment and its influence on diabetes is documented by Hipp and Chalise (9) in “Spatial Analysis and Correlates of County-Level Diabetes Prevalence, 2009–2010.” These articles provide further evidence of how exposure to poor-quality physical and social environments may place populations at risk for developing obesity and diabetes.

Six articles examine the effect of environmental settings and policies on diabetes and obesity outcomes. The perception of the quality of the home and school settings and its influence on eating behavior is assessed by Clarke et al (10) in “Influence of Home and School Environments on Specific Dietary Behaviors Among Postpartum, High-Risk Teens, 27 States, 2007–2009.” Three articles describe developing research on interventions addressing the influence of income in workplaces: “Enhancing Workplace Wellness Efforts to Reduce Obesity: A Qualitative Study of Low-Wage Workers in St Louis, Missouri, 2013–2014” by Strickland et al (11), “Worksite Influences on Obesogenic Behaviors in Low-Wage Workers in St Louis, Missouri, 2013–2014” by Strickland et al (12), and “Review of Measures of Worksite Environmental and Policy Supports for Physical Activity and Healthy Eating” by Hipp et al (13). Two articles describe the influence of policy on diabetes prevention and control. Brown and McBride (14) analyze the state of being uninsured and its influence on health care use in “Impact of the Affordable Care Act on Access to Care for US Adults With Diabetes, 2011–2012.” Purnell et al (15) discuss the importance of linking approaches across settings to address diabetes prevention and control: “Outside the Exam Room: Policies for Connecting Clinic to Community in Diabetes Prevention and Treatment.”

Two articles examine innovations in health communication science and technologies for addressing diabetes disparities. Harris et al (16) describe emerging evidence on the use of social media and crowdsourcing to influence diabetes-related behavior in “Diabetes Topics Associated With Engagement on Twitter.” Caburnay et al (17) address the use of mobile technologies in “Evaluating Diabetes Mobile Applications for Health Literate Designs and Functionality, 2014.” These articles explore innovative technologies that hold promise for overcoming communication barriers in reaching diverse audiences.

Sanders Thompson et al (18) review cultural adaptations of diabetes interventions and discuss the need for inclusion of cultural elements unique to racial/ethnic populations in “Use of Culturally Focused Theoretical Frameworks for Adapting Diabetes Prevention Programs: A Qualitative Review.” The review provides a road map for identifying key practices in adapting culturally appropriate, evidence-based interventions to real world settings.

Although these articles vary in topic and scope, the authors are consistent in their pursuit of better understanding the multilevel, multisector influences on the physical and social environment and how these environments affect behavior, health, and translational interventions to prevent diabetes. Transdisciplinary approaches such as those represented here are needed to recognize the broader causes of disparities, better inform actions to mitigate the effect of these root causes of disease, and promote sustainable progress in preventing diabetes in diverse populations. Opportunities that encourage the integration of diverse perspectives can lead to transformational research and sustainable impact.
